# DOMMINO 2.0: integrating structurally resolved protein-, RNA-, and DNA-mediated macromolecular interactions

**DOI:** 10.1093/database/bav114

**Published:** 2016-01-30

**Authors:** Xingyan Kuang, Andi Dhroso, Jing Ginger Han, Chi-Ren Shyu, Dmitry Korkin

**Affiliations:** ^1^Informatics Institute, University of Missouri, Columbia, MO, USA,; ^2^Department of Computer Science and Bioinformatics and Computational Biology Program, Worcester Polytechnic Institute, Worcester, MA, USA,; ^3^Department of Electrical and Computer Engineering,; ^4^Department of Computer Science, University of Missouri, Columbia, MO, USA

## Abstract

Macromolecular interactions are formed between proteins, DNA and RNA molecules. Being a principle building block in macromolecular assemblies and pathways, the interactions underlie most of cellular functions. Malfunctioning of macromolecular interactions is also linked to a number of diseases. Structural knowledge of the macromolecular interaction allows one to understand the interaction’s mechanism, determine its functional implications and characterize the effects of genetic variations, such as single nucleotide polymorphisms, on the interaction. Unfortunately, until now the interactions mediated by different types of macromolecules, e.g. protein–protein interactions or protein–DNA interactions, are collected into individual and unrelated structural databases. This presents a significant obstacle in the analysis of macromolecular interactions. For instance, the homogeneous structural interaction databases prevent scientists from studying structural interactions of different types but occurring in the same macromolecular complex. Here, we introduce DOMMINO 2.0, a structural Database Of Macro-Molecular INteractiOns. Compared to DOMMINO 1.0, a comprehensive database on protein-protein interactions, DOMMINO 2.0 includes the interactions between all three basic types of macromolecules extracted from PDB files. DOMMINO 2.0 is automatically updated on a weekly basis. It currently includes ∼1 040 000 interactions between two polypeptide subunits (e.g. domains, peptides, termini and interdomain linkers), ∼43 000 RNA-mediated interactions, and ∼12 000 DNA-mediated interactions. All protein structures in the database are annotated using SCOP and SUPERFAMILY family annotation. As a result, protein-mediated interactions involving protein domains, interdomain linkers, C- and N- termini, and peptides are identified. Our database provides an intuitive web interface, allowing one to investigate interactions at three different resolution levels: whole subunit network, binary interaction and interaction interface.

**Database URL:** http://dommino.org

## Introduction

Interactions between three major types of macromolecules in a cell, proteins, DNAs and RNAs, underlie the cell’s basic functioning and are implicated in many diseases (1–5). These diverse molecular interactions also constitute the basic building blocks of a complex macromolecular assembly (6–8). While, some of the macromolecular assemblies involve interactions of only one type, structures of other molecular assemblies, such as ribosome or RNA polymerase complex, are a product of an elaborate interplay of macromolecular interactions mediated by both proteins and nucleic acid molecules (9, 10). Understanding structures of macromolecular interactions at the atomic level could help to provide insights into the function and evolution of the constituting macromolecules and, ultimately, overall macromolecular assemblies.

Throughout the last decade, there have been a growing number of databases on structurally resolved macromolecular interactions. Most, if not all, of these databases have used RCSB Protein Data Bank (PDB) (11) and Protein Quaternary Server (PQS) (12) as sources for structural information. The databases can be grouped into three major classes based on their macromolecular content. The protein-protein interaction databases, including 3DComplex (13), 3DID (14), DOMINE (15), DOMINO (16), INstruct (17), iPfam (18), PepX, (19), PIBASE (20), PSIBASE (21), SCOPPI (22), SNAPPI-DB (23), the first version of DOMMINO (24) and others (25), mainly focus on domain-domain interactions and less frequently on the interactions of other types, such as protein-peptide, protein-termini, or domain-linker mediated interactions. Many of the above databases use the sequence- and structure-based classification of the interacting domain based on SCOP (26), CATH (27), PFAM (28) and other domain definitions. Databases on RNA- and DNA-mediated interactions occur in much smaller numbers and include BIPA (29), NDB (30), NPIDB (31) and PRIDB (32). These databases contain primarily the structurally resolved protein–DNA and protein–RNA interactions.

The three key features most databases lack include the integration of all macromolecular interaction types into a single relational database, the frequent and automatic updating mechanism and on-the-fly classification of the newly released PDB structures without waiting for a new release of the corresponding sequence-based or structure-based annotations. Perhaps most underrepresented in the current databases are the macromolecular interactions of the unstructured (disordered) regions, such as interdomain linkers, C- and N- termini, with other proteins, DNA and RNA molecules. There is a growing volume of evidence that the unstructured regions play crucial functional roles and have been implicated in a number of biological processes (33–35). Terminal residues are involved in interactions with multi-domain scaffold proteins (36, 37) as well as single- and double-stranded DNAs (38, 39); relevant functions also include protein sorting, signaling, translation termination and ubiquitination-induced degradation (34). Interdomain linkers have also been found linked to a wide range of signaling processes, allosteric communication, transcription and other functions that involve linker–protein, linker-DNA and linker-RNA interactions (35, 40–42). Datasets of unstructured regions have been previously collected and analyzed (40, 43–45), however similarly comprehensive collections of the macromolecular interactions mediated by these regions are yet to be published. Other features that some macromolecular interaction databases lack and others provide include flexible visualization of the binary and higher order interactions at the different resolution levels, as well as extraction of the key information on interactions, such as binding sites, interface contact residue pairs, and atomic coordinates.

In this work, we present DOMMINO 2.0, a database on structurally resolved macromolecular interactions. The database integrates interactions mediated by all three types of biological macromolecules, DNA, RNA and proteins. In the Methods section, we describe the basic data sources and data processing steps, following by the description of the subunit annotation procedure, and definitions for residue-based and nucleotide-based macromolecular interactions. We then talk about the basic interaction statistics on the current version of DOMMINO 2.0 and describe a web interface for the interaction data retrieval. Finally, we describe two large-scale case studies, where the data from DOMMINO 2.0 are used to obtain three-dimensional macromolecular interaction networks: a human macromolecular interactome and human–virus interactome.

## Methods

Building a comprehensive database system on macromolecular interactions requires a systematic annotation of basic structural subunits that are potential interacting partners. The subunit definitions, in turn, will allow dissecting a macromolecular complex into a set of binary interactions. DOMMINO 2.0 includes definitions of eight subunit types. Specifically, we categorize the protein subunits as: (i) domain, (ii) interdomain linker, (iii) C-terminal region, (iv) N-terminal region, (v) peptide and (vi) undefined polypeptide chain. Then, we add two nucleotide subunits: (vii) DNA subunit and (viii) RNA subunit. To facilitate investigations of the relationships among nucleotide- and protein-mediated macromolecular interactions in homologous complexes and across multiple organisms, we also integrate into DOMMINO 2.0 the corresponding organism, molecule type, evolutionary classification and other basic biological information. Reconstruction of the macromolecular interaction network for each PDB structure allows us to study the complexity of interactions within complex macromolecular assemblies. Finally, to handle the rapidly growing size of the database and to expedite the data retrieval process, DOMMINO 2.0 benefits from an optimized architecture and has been implemented in the Oracle database system.

### Data sources and preprocessing

**Figure 1.**
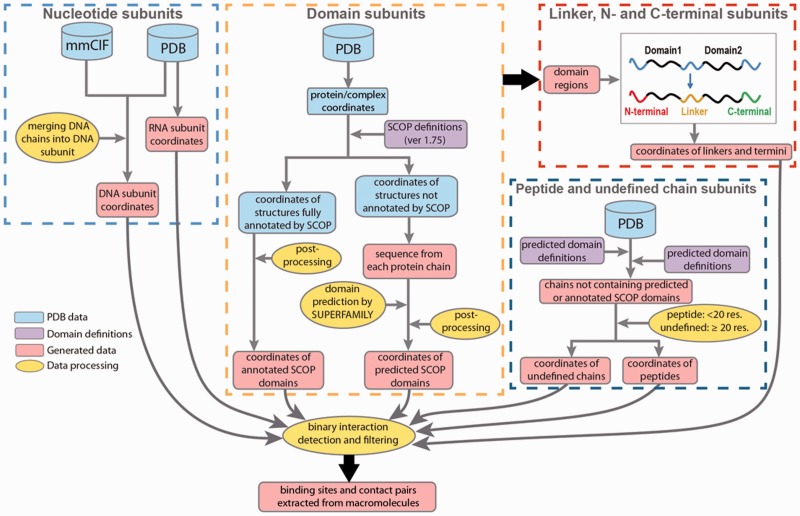
Data processing in DOMMINO 2.0. Eight subunit types are identified in four processing stages: (1) nucleotide subunits, including DNA and RNA subunits, (2) domain subunits, (3) unstructured subunits, including interdomain linkers, C-termini and N-termini and (4) peptides and undefined chain subunits.

DOMMINO 2.0 leverages several databases and tools to collect and process macromolecular interaction data (Figure 1). The structural information is first extracted from The Protein Data Bank (PDB) (11) during the weekly automated updates; the collected PDB files are used to extract 3D coordinates and biological information. For our initial pool, we consider any PDB file containing more than one chain. Then, we complement this pool with another set consisting of single-chain PDB files with more than one macromolecular subunit identified. The set provides additional candidates for intra-molecular interactions. The base pair information is obtained from the data item _ndb_struct_na_base_pair. pair_name of the PDB data dictionary and macromolecular Crystallographic Information File (mmCIF) (46). Using the latter format allows annotating individual nucleotide chains that constitute a DNA molecule.

Next, to annotate the protein subunits, we rely on a hierarchical structural classification of proteins (SCOP), an accurate, manually curated annotation system (26). The basic hierarchy of SCOP includes four levels: Class, Fold, Superfamily and Family. Our database employs the Family classification level. At this level, structural subunits typically share similar structure and sequence as well as functional properties, and are homologs. The most recent publicly released version of SCOP domain annotation (1.75) has been utilized to identify the domain subunits in the polypeptide chains constituting a PDB entry. However, since the most recent public release of SCOP in 2009, more than 66.6% of PDB entries (74 498) have not been annotated by SCOP. For those PDB structures, we use a Hidden Markov Model based prediction method, SUPERFAMILY, that locates positions of all structurally defined domains and classifies them by SCOP (47). Finally, we use the ASTRAL compendium to define peptide subunits (48).

## Annotation of basic macromolecular subunits

### Nucleic acid subunits

In DOMMINO 2.0, two new subunit types have been introduced: DNA chains and RNA chains. The structural organization of DNA-based complexes found in PDB is quite diverse and includes not only a typical double helix structure, but structures with four and more nucleotide chains. To identify a single DNA subunit, the DNA complexes are broken down into chains using the following procedure: we first identify all DNA chains and then select each DNA chain with at least one base pair being complimentary paired with a base pair of another DNA chain. In total, 4,844 PDBs containing DNA subunits were annotated. Interestingly, many DNA-containing macromolecular complexes include 3, 4, and higher numbers of DNA chains. For instance, Flp recombinase-Holliday junction complex (PDB ID: 1FLO) is comprised of 8 DNA chains.

RNA molecules are defined in a more straightforward way: we treat each RNA chain as one macromolecular subunit. In addition to the RNA and DNA chains, we have detected 160 hybrid nucleotide chains, where each hybrid chain contains both DNA and RNA bases. However in this version, the interactions involving hybrid nucleotide chains were not considered.

### Protein subunits

The protein subunits are assigned through a series of annotation steps. First, for each protein chain in a PDB file, we map all domains subunits annotated by a SCOP family. To do so, we first use manually annotated SCOP domain definitions retrieved from file *dir.cla.scop.txt* in the SCOP Parseable Files directory. The most recent version of SCOP 1.75 is used to annotate 110 800 SCOP *domains* from 38 221 PDB entries. However, PDB is being constantly updated, and some of the previously deposited PDB files have been modified. As a result, we have found that a number of SCOP domains cannot be correctly located in the current versions of some PDB entries, and we can thus use only 107 360 SCOP domains from 37 439 PDB entries.

Next, we scan the unannotated protein regions using SUPERFAMILY tool (47) to predict SCOP domains. For our predictions, we use threshold of E ≤ 0.01, reflecting similarity between the target protein sequence and the hidden Markov models. Using this protocol, we have predicted 238 180 additional SCOP domains.

Even after both annotation protocols, SCOP-based and SUPERFAMILY-based, are used, there are still long protein regions and even entire protein chains that do not have any SCOP domains assigned to them. In a case when the entire protein chain does not contain a single annotated subunit, we classify it as a *peptide* if the chain length is < 20 residues, following the ASTRAL definition (48), and as an *undefined chain* otherwise. The undefined chain is likely to correspond to a novel protein domain or several domains. If a protein chain contains one or more annotated SCOP domains, we will further annotate the unannotated regions. An unannotated region is called a *domain linker* if it is located between two SCOP domains (Figure 1); a *C-terminal* if there is a domain located to the left of the region, but no domain is located to the right; and *N-terminal* if a SCOP domain is located to the right but there is no domain on the left hand side. The latter three types of the regions represent the unstructured regions.

### Determining macromolecular interaction interfaces and subunit networks

A macromolecular interaction formed by two subunits of any of the eight types is defined using residue-residue, residue-nucleotide, or nucleotide-nucleotide contact definitions. In total, 36 interaction types mediated by eight different subunits types are defined and analyzed (Table 1). Once all binary interactions are defined for a PDB file, the full subunit interaction network is determined and visualized.

**Table 1.** Data statistics

	D	C	N	L	U	P	DNA	RNA
D	412,658	213,400	187,608	75,360	15,146	13,592	9,508	28,905
C		9,480	25,639	5,893	2,726	849	327	3,199
N			7,690	4,600	2,695	499	501	5,268
L				3,681	475	265	225	70
U					19,656	834	295	2,297
P						1,371	63	54
DNA							714	245
RNA								2,951

The distribution of macromolecular interactions in DOMMINO 2 (September, 2015) across 36 interaction types mediated by eight different subunits types: domains (D), C-termini (C), N-termini (N), inter-domain linkers (L), undefined chains (U), peptide (P), DNA molecules (DNA) and RNA molecules (RNA).

A *protein–protein interaction* is defined through detecting the residue*–*residue contact pairs. To identify all residue*–*residue contact pairs between two protein subunits, we calculate the shortest distance between every pair of residues, one residue from each subunit. Here, the shortest distance between two residues is, in turn, defined as the smallest Euclidean distance between a pair of heavy atoms, one atom from each residue. A pair of residues is defined as a residue-residue contact pair if the shortest distance between them is no more than 6 Å, a widely adopted threshold (49–52). Moreover, the user can specify a minimal number of contact pairs that defines a protein*–*protein interaction. The default threshold is 10 contact pairs. Finally, there is an additional criterion to define the intra-chain interactions between the sequentially adjacent subunits. Specifically, to avoid the false positives when detecting contact pairs between a residue at the C-terminal of the first subunit and a residue at the N-terminal of the second subunit due to their sequential proximity, we require the minimal sequential distance for residues from the adjacent subunits to be more than 10 residues.

*Protein–DNA and protein–RNA interactions* are defined through the residue*–*nucleotide contact pairs. The definition of a residue*–*nucleotide contact pair differs from that one of a residue*–*residue contact pair due to the differences in the non-covalent bonds frequently occurring in the corresponding types of macromolecular interactions (53–55). Specifically, to detect a residue-base contact pair, we use the maximum distance threshold of 3.5 Å calculated between the oxygen and nitrogen atoms in the nucleic base and a heavy atom in the protein residue. *DNA–DNA, DNA–RNA*, and*RNA–RNA interactions* are defined similarly to the previous interaction types and use the distance threshold of 3.5 Å between the nitrogen-nitrogen or oxygen*–*nitrogen atom pairs for the interacting DNA and/or RNA subunits.

## Database content

DOMMINO 2.0 provides a comprehensive set of macromolecular interactions mediated by proteins, DNA and RNA molecules together with the corresponding sets of contact pairs and binding sites extracted from the binary interfaces. The interaction data and the relevant information in DOMMINO 2.0 are organized into a relational database with support for a web-based search and retrieval. The database is updated through an automated protocol for synchronization with the most recent release of PDB. Specifically, DOMMINO 2.0 and the corresponding file system are automatically updated on a weekly basis, which follows the PDB’s weekly release schedule. While most updates include newly added interactions, sometimes some of the previously existing entries may be removed in a new PDB release because they are erroneous or outdated. This, in turn, requires removal of that entry from DOMMINO 2.0. The PDB log file that records the deleted and newly added entries is used to flag the deleted entries in the database. If the PDB ID of a newly added PDB entry does not exist in DOMMINO 2.0, the entire four-stage processing procedure (Figure 1) is applied for this PDB entry to extract the interaction data. Alternatively, in case when the PDB entry is present in the database, we first remove from DOMMINO 2.0 the old PDB entry and related data, and then add a new entry with the same PDB ID.

Currently, DOMMINO 2.0 database consists of 19 tables and occupies 3.2 GB of space, not counting the coordinate files. Compared with the previous version of the database, DOMMINO 2.0 is now implemented in the Oracle Enterprise databaase system, as opposed to MySQL, allowing our database to support and scale a much large number of interactions. The database has been also fully restructured to deliver faster query retrieval time. For instance, the retrieval time for all domain-domain interactions has now decreased from 0.29 to 0.11 s.

**Figure 2.**
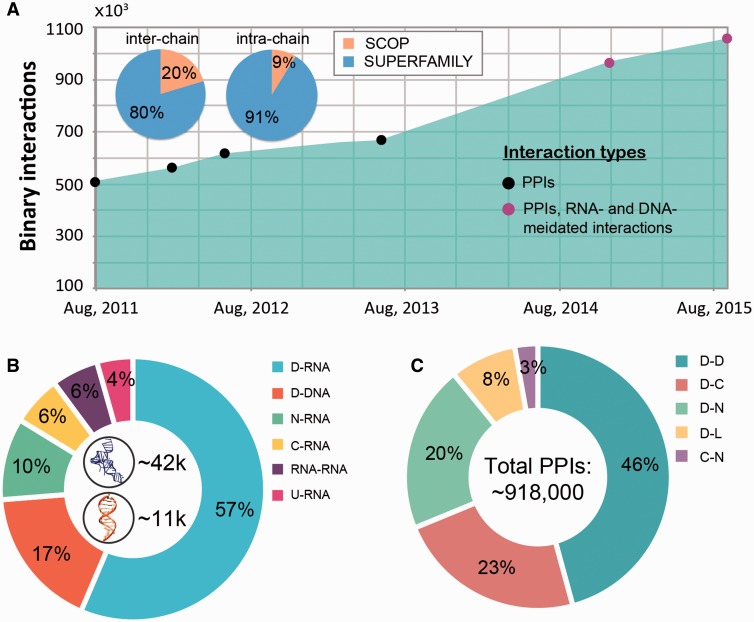
Interaction statistics for DOMMINO 2.0. (**A**) The growth of the number of binary macromolecular interactions in DOMMINO, from DOMMINO 1.0 (interactions between two polypeptide subunits only) to DOMMINO 2.0 (polypeptide-, DNA- and RNA-mediated interactions, shown in circles). The majority of interactions involving protein subunits are annotated using SUPERFAMILY (82% of inter-chain and 92% of intra-chain interactions). (**B**) Relative contribution of six most abundant interaction types involving a nucleotide subunit. Shown in the center are the total numbers of RNA- and DNA-mediated interactions. (**C**) Relative contribution of five most abundant polypeptide-polypeptide interaction types. Denoted are eight different subunits types: domains (D), C-termini (C), N-termini (N), inter-domain linkers (L), undefined chains (U), peptide (P), DNA molecules (DNA) and RNA molecules (RNA). Shown in the center is the total number of polypeptide-polypeptide interactions (PPIs).

The macromolecular interactions in our database include both, intra-species and inter-species interactions. The inter-species interactions represent the minority of all interactions (only ∼2.5%, see Figure 2A) and include 19 987 protein*–*protein interactions and 5397 nucleic acid mediated interactions (as of September, 2015). The interactions come from over 4700 species across all three domains of life (*Archaea*, *Bacteria* and *Eukaryota*) and viruses. While both DNA- and RNA-mediated interactions are exclusively inter-chain interactions (Figure 2B), protein-protein interactions can be both inter- and intra-chain. Currently protein–protein interactions constitute the majority of interactions (1 004 117 interactions, 94.8% of all interactions) in DOMMINO 2.0 with domain–domain interactions being the most populated interaction type (412 658 interactions, 39.0% of all interactions). The largest subset of the total of 51 212 residue–nucleotide interactions corresponds to protein domain-RNA interactions (28 905 interactions, 2.7% of all interactions), while the largest subset of the total of 3,910 nucleotide-nucleotide interactions corresponds to RNA–RNA interactions (2,951 interactions, 0.3% of all interactions).

The analysis of the data has revealed several interesting trends. First, we find that the number of interacting protein domains that are annotated by SUPERFAMILY is significantly higher than that of SCOP annotated domains (813 638 *vs.* 142 59 interactions). Even more importantly, we have observed a significantly faster growth of the number of interactions whose subunits are SUPERFAMILY- annotated. Second, the statistical analysis reveals that the domain-domain interactions are no longer the most populated class of structurally resolved macromolecular interactions. Instead, the interactions between the unstructured regions and domains constitute the biggest part of DOMMINO 2.0: 412 658 interactions between a pair of protein domains versus 505 106 interactions between a domain and an unstructured region (linker, C- or N-terminus, or peptide) with the interactions between domains and C-termini being the most prevalent ones (213 400 interactions).

Structures of the individual subunit-subunit interactions of all 36 types are available from DOMMINO 2.0. In addition, species name, molecule name, UniProt accession number and SCOP domain classification are deposited in our relational database. In order to provide the most current macromolecular interaction information, DOMMINO 2.0 is automatically updated and is synchronized to follow the PDB’s weekly updates.

## User interface

**Figure 3.**
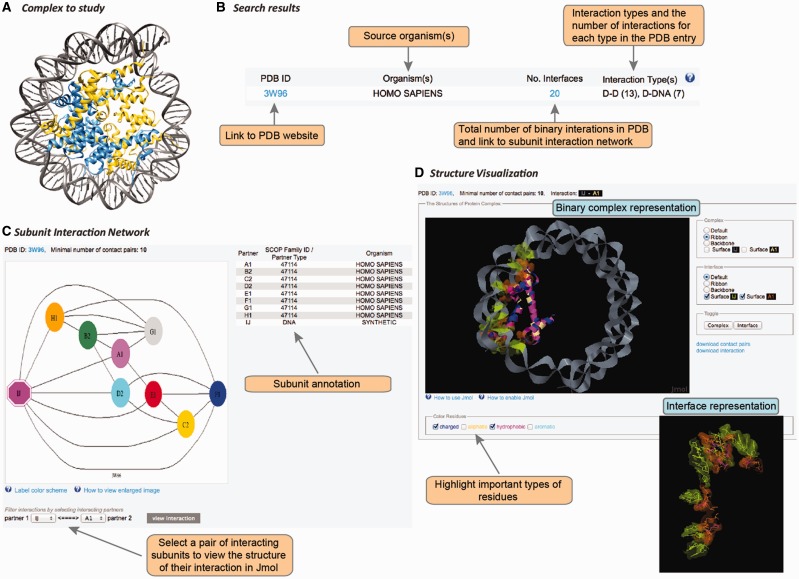
User interface in DOMMINO 2.0. User interface consists of three main components allowing the user to study the complex of interest at the subunit network, binary interaction or interaction interface levels. (**A**) Each macromolecular complex deposited to PDB is retrieved and analyzed in DOMMINO 2.0 due to the database’s weekly updates that follow updates of PDB. (**B**) Basic and advanced search allows the user to retrieve a complex or group of complexes of interest. (**C**) Subunit network view allows investigating all inter-subunit interactions for a selected complex and specifying a binary interaction for in-depth study. (**D**) Visualization of a binary interaction provides the whole-interaction and interface-only views. The user can highlight important types of residues as well as download the interface contact pairs and interaction coordinates.

DOMMINO 2.0 features an updated web interface that allows studying the interplay between protein-, RNA- and DNA-mediated interactions in macromolecular complexes (Figure 3). The interface is designed to facilitate the search, retrieval, analysis and visualization of the macromolecular interactions within a macromolecular assembly at different resolution levels, from the subunit interaction network to all-atomic representation of the individual interactions.

### Search and retrieval of macromolecular complexes

The interaction data can be retrieved and analyzed using the web-server’s simple search query, advanced search query, and browsing mode. The simple search query allows the user to retrieve a specific macromolecular complex using its PDB ID (Figure 3A and B). If the queried PDB ID corresponds to a macromolecular structure with at least one subunit-subunit interaction, this structure will be retrieved and represented as a subunit-level interaction network (see Subunit-level visualization of the interaction network section).

The advanced search query is more flexible than the simple search query, since it may include (through logical AND) multiple search fields. We have currently implemented four types of the search fields: interaction type, organism, SCOP family and keyword. The interaction type field offers the possibility of searching any of the 36 subunit-subunit interactions types. For instance, ‘D-N’ corresponds to an interaction between a domain and an N-terminal, while ‘RNA-L’ corresponds to an interaction between an RNA molecule and a linker. The organism field corresponds to the organism name and can be entered either as a taxonomic name (e.g. *Homo sapiens*) or a common name (e.g., human). Searching using the SCOP family field requires specifying a SCOP ID. Finally, the keyword field allows searching through the keywords provided in a PDB file and concerned with a description of the macromolecular complex or any of its components. When there are more than 50 PDB IDs retrieved, the user can either refine the query or retrieve the results as a compact list of PDB IDs. When a search query results in < 50 PDB IDs, the server will show the query results as a more detailed list that includes the number of binary interfaces constituting the macromolecular complex, types of macromolecular interaction and the source organisms.

Another way of retrieving a macromolecular interaction is by using the browsing mode. In this mode, all interactions are hierarchically organized first based on the interaction type, and then, where it is possible, based on the SCOP families of interacting subunits. All SCOP families are then further grouped based on SCOP classes and folds.

### Subunit-level visualization of the interaction network

For each PDB entry, the overall intra-complex subunit interaction network is annotated and visualized. The network is represented as a graph, where the nodes are shape- and color-coded (Figure 3C). In total, there are eight shapes, each corresponding to a protein subunit type, RNA or DNA. Subunits of the same color are located within the same chain. The complete information on the shapes and colors is provided through a pop-up help window. Each pair of nodes is connected by an edge if the corresponding subunits are found to interact after the threshold of the minimal number of contact pairs (either default or provided by the user) is applied. Thus, a subunit that shares at least one pair of contact residues with another subunit but not passing the minimal number of pairs threshold, will be shown disconnected. The label of each subunit consists of a chain letter and the order number of this subunit in the chain. The network is accompanied with a table describing subunit details such as subunit label, SCOP family for the domain subunit or other subunit types, and the source organism. The user can then select a pair of interacting subunits for further structural studies.

### Atomic-level visualization of binary macromolecular interactions

Once a pair of chains from an interaction complex is selected, the user is able to study structure of this binary interaction at the atomic level (Figure 3D). This visualization stage is implemented using JMol tool, an open-source molecular visualization software supported by the community and freely available at www.jmol.org (56). The visualization of the binary interaction is flexible. First, the user can visualize either the overall complex or just the interaction interface. Second, either complex or interface can be visualized using one of four modes of representation: ball-n-stick, ribbon, backbone or surface. Third, the user can highlight several types of residues in the interface that may play important roles in the interaction: charged, aliphatic, hydrophobic and aromatic. We note that the user can change all the above representation parameters on the fly. Finally, for each visualized binary interaction, the user is able to download (i) the coordinates of the binary complex in the PDB format extracted from the overall PDB file and (ii) the list of contact residue pairs constituting the interface.

### Help and tutorial

DOMMINO 2.0 is designed to provide help to the user in two different formats. First, all functional elements in the web-server (e.g. buttons, links, text boxes, *etc*.) are accompanied by a pop-up help window, providing the details about the functional element and its possible options. Second, we designed step-by-step tutorial showing how to perform the basic and advanced searches, navigate through the results and visualize the individual complexes and binary interactions.

## Application to comparative interactomics

DOMMINO 2.0 provides a unique capability to work with the interactions across all three basic types of macromolecules on the whole-system scale. To demonstrate that, we have analyzed two 3D-resolved interactomes: a human family-centered macromolecular interactome, and a human-viral interactome.

**Figure 4.**
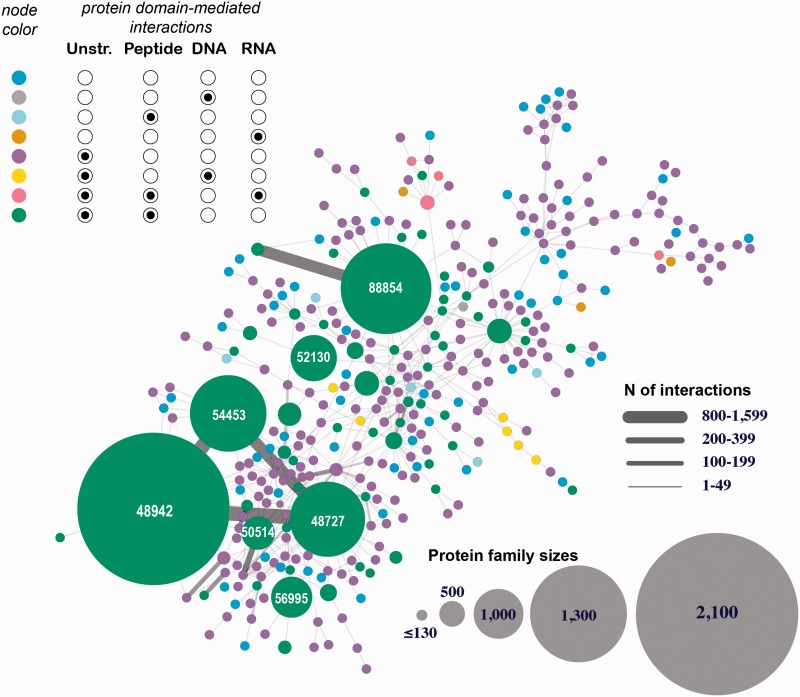
Human 3D interactome that includes RNA-, and DNA-mediated interactions. Shown is the largest connected component comprised of 39 399 protein subunits annotated into 354 SCOP protein families and mediating 26 516 protein–protein interactions and 257 protein–nucleotide interactions. The nodes correspond to protein families and are colored based on the types of interactions mediated by the proteins. Unstr corresponds to all unstructured protein subunits (C- and N-termini, and domain linkers). The numbers shown in the large nodes correspond to the SCOP IDs for those large protein families. For example, 48942 is a SCOP ID for the ‘C1 set domains (antibody constant domain-like)’ SCOP family of immunoglobulins.

The first interactome is constructed through integrating all eight types of structurally resolved human interactions that involve at least one protein subunit associated with a SCOP family (Figure 4 depicts the largest connected component of this network). The analysis of the obtained network showed the presence of large sets of protein families connected with each other through macromolecular interactions mediated by the individual family members. This finding suggests that the current structural coverage of the human interactome has reached the point where system-wide studies at the atomic resolution are possible. In addition, we find that some protein families are presented exclusively with specific types of interactions (e.g., proteins SCOP family 46789: LexA repressor are involved predominantly with protein–DNA interactions that are structurally resolved), while other families include interactions of multiple types (e.g. family 53017: lambda exonuclease). Even more importantly, large connected subnetworks include a significant number of DNA- and RNA-mediated interactions, connections that are excluded in traditional protein-protein interaction networks.

**Figure 5.**
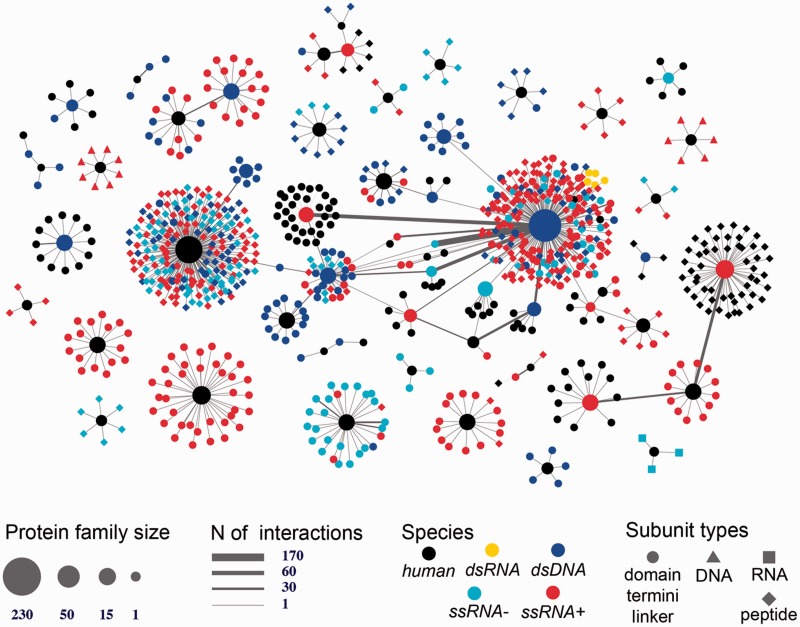
Human-viral inter-species 3D interactome. Shown is the entire human-virus macromolecular interactome including 1070 subunits such as inter-domain linkers, peptides, protein termini, and protein domains annotated into 104 SCOP families. The subunits mediate 1865 host–pathogen protein–protein interactions and 17 protein-nucleotide interactions. The nodes are shaped based on the macromolecular type and are colored based on the species type (either human or viral family species).

For the second network, we have extracted from DOMMINO 2.0 all macromolecular interactions between human and viral macromolecules and use a similar visualization, with the virus macromolecular nodes color-coded and grouped according to the Baltimore classification (Figure 5). The 3D structural human–viral network provides an important insight about the nature of host–pathogen interactions. First, several human proteins, RNAs and DNAs are found targeted by multiple viruses in the network. Furthermore, the reverse phenomenon, where same viral protein targets different host macromolecules, is also observed. Both facts have been previously documented in a study that was limited to protein-protein interactions (57). Thus, our database allows expanding this analysis to the macromolecular interactions involving the RNA and DNA molecules.

## Conclusion

The newly designed database of structurally resolved macromolecular interactions, DOMMINO 2.0, contains comprehensive information about the interactions mediated by all three major classes of biomolecules in a living organism: proteins, RNAs and DNAs. The database’s web interface allows the user to search and study interactions at the different resolution levels, from investigating the subunit interaction network of a whole macromolecular assembly to studying atomic details of a specific interaction interface formed between two macromolecules. Structural classification of the interacting subunits implemented in DOMMINO 2.0 has made this database a useful tool in comparative interactomics analyses involving multiple types of macromolecules.

The two applications presented in this work have shown the possibilities of utilizing the structural interaction data from DOMMINO 2.0 on the large-scale, by constructing structurally resolved intra- or inter-species macromolecular interaction networks. One should use caution, however, when making biological conclusions from such a network. First, it is likely to be incomplete due to the incomplete (and sometimes biased) coverage of the interactome in PDB. Second, the network might contain interactions that are not physiological, e.g., interactions that are crystallographic artifacts or that are obtained from artificial constructs. The first problem can be addressed through modeling structures of prospective interactions (58), while second problem can be addressed through filtering out the non-physiological interactions using computational methods (59, 60), which we plan to implement in the next version of DOMMINO.

The key difference from the previous version (1.0) of DOMMINO is the integration of nucleotide-mediated interactions into the database and its web-based search, retrieval, and interaction visualization. Another important difference is the streamlined architecture of the database, which is now implemented in Oracle, instead of MySQL. The improvements have made the retrieval more than twice faster than in DOMMINO 1.0, in spite of the substantially increased size of the interaction dataset.

With the vast amounts of high-throughput structural data becoming available, the future development of the DOMMINO database will focus on expanding and cleaning the interaction data. First, we will include the macromolecule-ligand interaction information: recent studies have showed intriguing and potentially important interplay between the ligand binding and protein-protein interactions (61). Second, structural information on protein–protein interactions can be used to understand the functional impact of many genetic variants associated with various genetic disorders (62, 63). We will integrate into DOMMINO the genetic variation data associated with diseases and extracted from publicly available sources (64, 65). Finally, to allow the user control over the interaction data quality, we will implement several filters for detection of potential biological artifacts and redundant interactions.

## Supplementary Data

Supplementary data are available at *Database* Online. 

*Conflict of interest*. None declared.

Supplementary Data
