# Supplementary material for: DOMMINO 2.0: integrating structurally resolved protein-, RNA-, and DNA-mediated macromolecular interactions
Source: Database (Oxford). 2016 Jan 30;2016:bav114. doi: 10.1093/database/bav114 (PMC4733329; doi:10.1093/database/bav114)
Supplement: Supplementary Data [file supp_2016_bav114_index.html]

DOMMINO 2.0: integrating structurally resolved protein-, RNA-, and DNA-mediated macromolecular interactions — Supplementary Data 

# DOMMINO 2.0: integrating structurally resolved protein-, RNA-, and DNA-mediated macromolecular interactions

## Supplementary Data

files

- Supplementary Data - zip file
